# A novel improved high-gain quadratic DC–DC converter based on two synchronous power switches and a two-winding coupled inductor

**DOI:** 10.1038/s41598-026-50006-2

**Published:** 2026-04-24

**Authors:** Ali Paşaoğlu, Ali Nadermohammadi, Hamed Shams, Hamed Sedighnejad

**Affiliations:** 1 Engineering and Natural Science Faculty, Computer Engineering Dept, İstanbul Rumeli University, stanbul, Trkiye Iran; 2 Faculty of Electrical and Computer Engineering, University of Tabriz, Tabriz, 51666-16471 Iran; 3 Department of Electrical Engineering, Lahijan Branch, Islamic Azad University, Lahijan, Iran

**Keywords:** Energy science and technology, Engineering

## Abstract

This paper presents a novel quadratic high-gain DC–DC converter employing two synchronously operated power switches and a two-winding coupled inductor for efficient low-voltage to high-voltage power conversion. The proposed topology achieves a large step-up voltage gain through the combined action of a quadratic boosting structure and magnetic coupling, while maintaining operation at moderate duty cycles. The voltage conversion ratio can be flexibly adjusted by both the duty cycle of the active switches and the turns ratio of the coupled inductor, providing an additional degree of freedom in converter design. The converter operates with a continuous input current, reduced voltage stress across semiconductor devices, and common-ground configuration between input and output, making it suitable for renewable-energy applications such as photovoltaic and fuel-cell systems. In addition, soft-switching operation is achieved for diode D_3_, which contributes to lower switching losses and reduced electromagnetic interference. The operating principles of the converter in continuous conduction mode are comprehensively analyzed, and the steady-state characteristics are derived. A detailed comparison with existing high-gain topologies demonstrates the advantages of the proposed structure in terms of voltage gain, component count, and device stress. Finally, experimental results obtained from a 300 W laboratory prototype operating at 50 kHz verify the theoretical analysis and confirm the capability of the converter to boost a 25 V input voltage to a regulated 400 V output with high efficiency.

## Introduction

Over the last decade, global energy policies have increasingly focused on reducing greenhouse gas emissions and expanding the contribution of renewable energy sources to electricity generation. As a result, renewable technologies are now widely viewed as viable alternatives to conventional fossil-fuel-based power systems. Among these resources, photovoltaic (PV) technology has gained particular attention due to the abundance of solar energy, its sustainability over long time horizons, and its relatively low environmental footprint^1^.

From a practical engineering standpoint, photovoltaic systems and fuel cells face a fundamental limitation due to their intrinsically low DC voltage output, which cannot be directly utilized in grid-connected systems or other high-voltage applications. Overcoming this constraint necessitates the use of high step-up DC–DC power conversion stages. Such converters boost the low-voltage DC generated by renewable energy sources to voltage levels suitable for downstream power processing and grid integration^2–6^. As a result, high-gain DC–DC converters have become indispensable components in contemporary renewable-energy infrastructures, enabling efficient clean-energy utilization and playing a vital role in global initiatives to curb air pollution, reduce environmental degradation, and alleviate climate change impacts associated with fossil-fuel dependence^7^. A variety of techniques have been developed to achieve high voltage conversion ratios, including switched-capacitor (SC) and switched-inductor (SL) approaches, active switched-inductor (A-SL) configurations, converters employing coupled inductors (CI), voltage-lift and voltage-multiplier (VM) topologies, as well as interleaved and cascaded multi-stage converter structures^8–24^. In many reported designs, these techniques are combined or hybridized to further enhance voltage gain and overall converter performance^25–30^.

Among the various high-gain configurations derived from these methods, quadratic converter topologies are particularly attractive due to their ability to achieve very large voltage conversion ratios. This characteristic arises from the presence of the (1 − D)^2^ term in the denominator of their voltage gain expression. In^31^, a quadratic boost converter combining SC and SL networks is proposed, achieving a relatively high voltage gain. However, because one of the capacitors is directly connected to the input source, the converter draws a pulsating input current, which is undesirable in many practical applications. A related topology introduced in^32^ also integrates SL and SC cells, employing two series-connected capacitors to regulate the output voltage. While this arrangement reduces capacitor voltage stress and ensures a continuous input current, the main switch is still subjected to a voltage stress equal to the output voltage. Although the converters in^32 and 32^ exhibit quadratic gain behavior, the high switch voltage stress leads to increased conduction and switching losses, making them unsuitable for very high-voltage applications.

In^33^, a group of quadratic converters combining coupled inductors with voltage-multiplier stages is proposed with the objective of reducing semiconductor voltage stress. Despite achieving high step-up ratios, these configurations suffer from substantial input current ripple, which limits their suitability for DC microgrid applications. Another quadratic topology reported in^34^ employs a CI with two cascaded boosting stages to obtain a high voltage gain; however, it experiences considerable power losses in the input diodes and exhibits a high input current.

A different quadratic structure with reduced device voltage stress is presented in^35^. Although it provides a high conversion ratio, the series connection between the CI primary winding and the input source results in significant input current ripple, placing additional stress on the renewable source. High-gain converters with lower input current ripple are proposed in^36] and [37^, where two- or three-winding CIs are combined with VM cells. These designs offer increased flexibility in adjusting the voltage gain but are constrained by high voltage stress on the main switch and large current stress on the input diodes.

Quadratic converters that integrate CIs with SC networks are introduced in^38–40^ to achieve high voltage gain. However, these configurations suffer from large peak currents flowing through the capacitors, leading to increased power losses and heightened electromagnetic interference. In^41^, a quadratic impedance-source converter combined with a CI is proposed, achieving high voltage gain with a relatively low number of components. Nevertheless, the semiconductor devices in this topology are still exposed to considerable voltage stress.

In^9^, a quadratic boost converter based on a SEPIC-derived stage is introduced, achieving a high voltage conversion ratio while ensuring low input current ripple—an essential requirement for DC microgrid applications. Nevertheless, the use of additional passive components, most notably the coupling capacitor, leads to increased resistive and reactive power losses, which adversely affect overall efficiency. Reference^42^ proposes a quadratic converter with soft-switching capability, continuous input current, and reduced voltage stress across the power devices. Although this approach successfully mitigates voltage stress, it employs a comparatively large number of components, particularly magnetic elements, resulting in diminished power density and increased system cost.

In light of the strengths and shortcomings of existing high step-up converter topologies, this work introduces a novel quadratic high-gain DC–DC converter. The proposed architecture provides two independent control variables for voltage regulation: the duty cycles of the active switches and the turns ratio of the coupled inductor. This additional design freedom enables enhanced optimization and flexibility. Unlike many conventional high-gain solutions, the proposed converter is not restricted to narrow duty-cycle operating regions and can function over the entire duty-cycle range from 0 to 1. Consequently, extremely high voltage gains can be realized even at moderate duty cycles, which contributes to lower conduction losses. Furthermore, the proposed topology imposes relatively low voltage stress on the MOSFETs, allowing the use of low-voltage-rated devices with reduced on-state resistance and, therefore, improved efficiency. The converter also ensures a continuous input current, making it well suited for interfacing with sensitive renewable energy sources such as photovoltaic systems. In addition, the use of two synchronously controlled active switches and a common ground between the input and output simplifies both control implementation and system integration. Finally, soft-switching operation is achieved for diode D_3_, effectively minimizing switching losses and suppressing electromagnetic interference.

### Proposed topology and operation modes

**Fig. 1:**
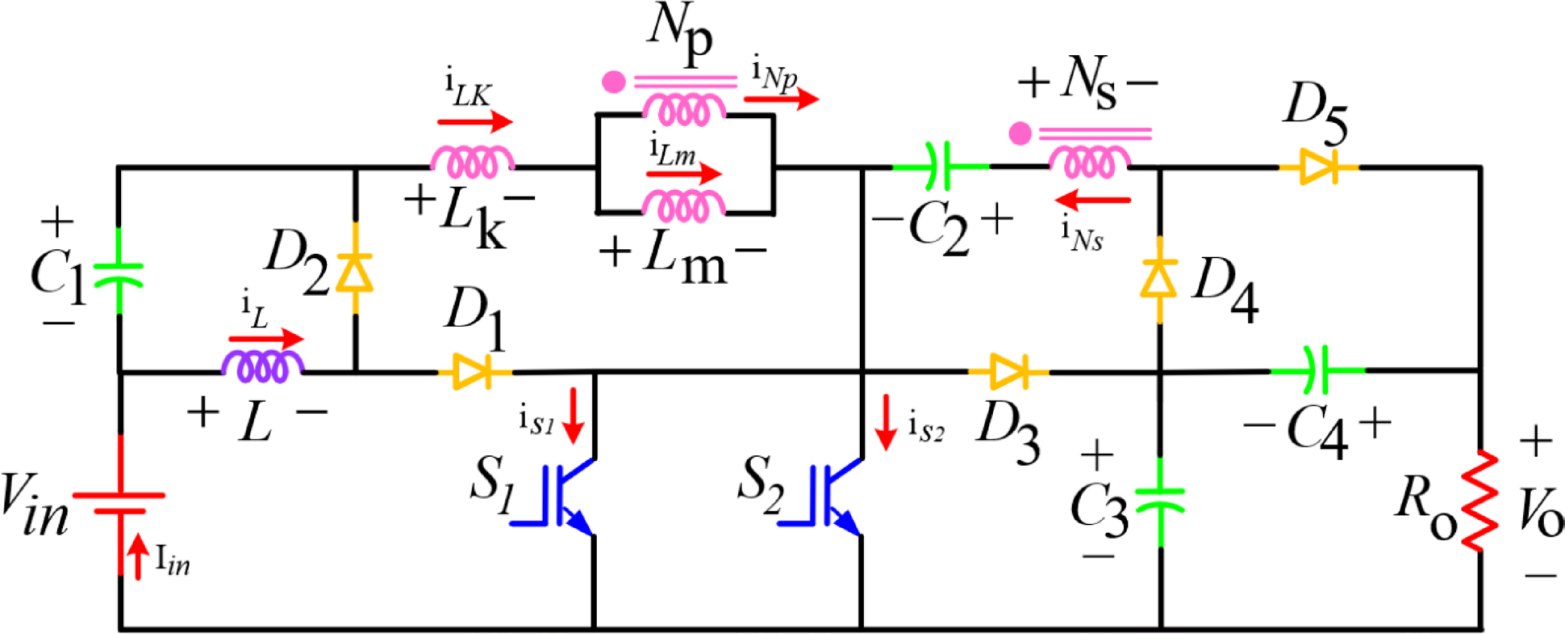
Suggested configuration.

**Fig. 2:**
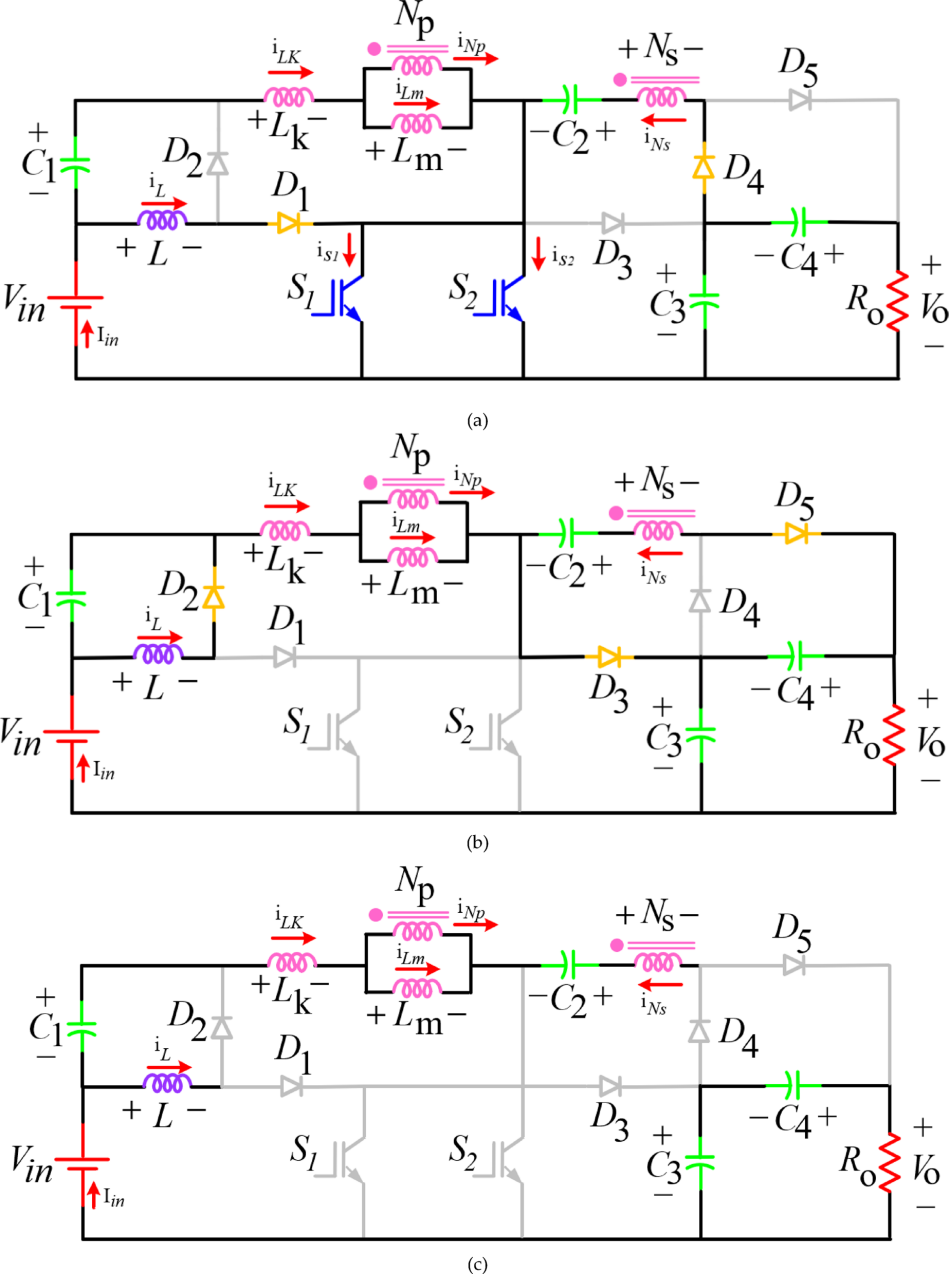
Equivalent circuits of the proposed converter: (a) Mode 1, (b) Mode 2, (c) Mode 3 (DCM).

**Fig. 3:**
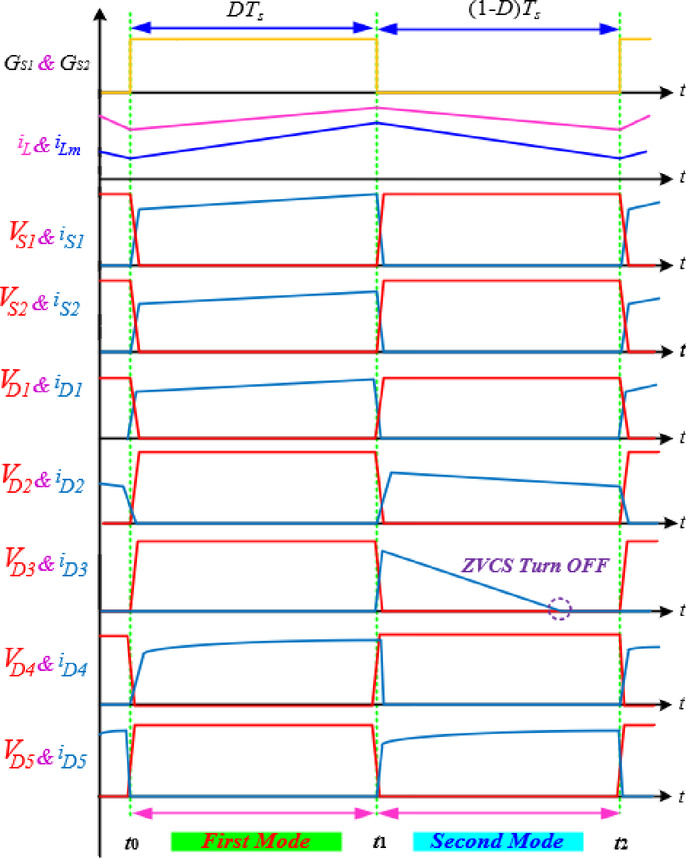
Main waveforms of the suggested structure.

Figure 1 illustrates the structural layout of the proposed converter topology. The proposed converter is primarily intended for applications that require efficient conversion from low-voltage renewable energy sources to standardized high-voltage DC buses. It is particularly suitable for photovoltaic systems, fuel-cell-based power units, and hybrid renewable-energy installations, where input voltages typically range from 12 V to 48 V while the required DC bus voltage is around 400 V. The 400 V output makes the converter directly compatible with DC microgrids, grid-connected inverter front ends, battery-energy storage systems, and electric vehicle DC links. In addition, the proposed topology can effectively supply high-voltage DC loads such as LED lighting systems and industrial DC infrastructures, benefiting from its high voltage gain and experimentally validated performance. The circuit comprises two controlled power switches (S₁ and S₂), four capacitors (C₁, C₂, C₃, and C₄), five diodes (D₁, D₂, D₃, D₄, and D₅), an input inductor (L), and a coupled inductor consisting of two magnetically coupled windings. These windings include a primary winding (Nₚ) and a secondary winding (Nₛ), with their relationship defined by the turns ratio n = N_s_/N_p_​. The strength of magnetic coupling between the windings is quantified by the coupling coefficient k = L_m_/(L_m_+L_k_). When the proffered high–voltage-gain converter operates in continuous conduction mode (CCM), each switching cycle can be divided into three distinct operating intervals, as depicted in Fig. 2. The corresponding voltage and current waveforms of the main circuit components during these intervals are presented in Fig. 3.

1 $${V_{Ns}} = n{V_{Lm}}$$

2 $${V_1} = {V_{Lm}} + {V_{Lk}} = \frac{{{V_{Lm}}}}{k}$$

3 $${V_{in}} = {V_L}$$

4 $${V_{C1}} = {V_1} - {V_{in}}$$

5 $${V_{Ns}} + {V_{C3}} = {V_{C2}}$$

6 $${V_O} - {V_{C3}} = {V_{C4}}$$

7 $${V_O} - {V_{C4}} + {V_{Ns}} = {V_{C2}}$$

8 $${I_L} - {I_{in}} = {I_{C1}}$$

9 $${I_{Lm}} = - {I_{Np}} - {I_{C1}}$$

10 $${I_L} = {I_{D1}}$$

11 $${I_{D1}} - {I_{S2}}+{I_{Lm}}+{I_{Np}}+{I_{C2}}={I_{S1}}$$

12 $${I_{Ns}}={I_{C2}}$$

13 $${I_{C3}}= - {I_O} - {I_{D4}}$$

14 $${I_{Ns}}={I_{D4}}$$

15 $${I_{C4}}= - {I_O}$$

16 $${I_{in}}={I_O}+{I_{C3}}+{I_{S1}}+{I_{S2}}$$

#### First mode

During this operating interval, both power switches are turned ON, causing diodes D₁ and D₄ to become forward-biased, while all other diodes remain in the blocking state. In this condition, the magnetizing inductance L_m_​ is excited through the conduction path formed by V_in_​–C₁–L_m_​–S₂. Consequently, the magnetizing current (i_Lm_​​) increases linearly, while capacitor C₁ releases energy along this path. Simultaneously, the input inductor L is energized via the V_in_​–L–D₁–S₁ loop, leading to a linear rise in the input current (i_L_​). Meanwhile, capacitor C₃ discharges and capacitor C₂ is charged through the conduction loop comprising C₃, D₄, the secondary winding N_S_​, C₂, and S₂. Capacitor C₄ discharges through the conduction path formed by C₃, C₄, and the load resistance R_O_​.Under these operating conditions, the governing equations for this interval can be derived as follows.

#### Second mode

During this operating interval, both power switches are turned OFF, causing diodes D₂, D₃, and D₅ to conduct, while the remaining diodes stay reverse-biased. In this mode, the currents through the input inductor L and the magnetizing inductance L_m_​ decrease approximately linearly as they release stored energy. The input inductor L is de-energized through the loop formed by L, D₂, and C₁, resulting in the charging of capacitor C₁. Simultaneously, the magnetizing inductance L_m_​ discharges and transfers energy to capacitor C₃ through the conduction path V_in_​–L–D₂–L_m_​–D₃–C₃. At the same time, capacitor C₂ releases its stored energy, while capacitor C₄ is charged via the loop consisting of V_in_​, L, D₂, L_m_​, C₂, the secondary winding N_S_​, D₅, C₄, and C₃. The voltage and current relationships governing this operating mode can therefore be expressed as follows.

17 $$- {V_L}={V_{C1}}$$

18 $$- {V_{in}} - {V_{C2}}+{V_1}+{V_{Ns}}+{V_O}={V_{C1}}$$

19 $$- {V_{in}} - {V_{C1}}+{V_1}= - {V_{C3}}$$

20 $$- {V_{in}} - {V_{C1}}+{V_1}+{V_O}={V_{C4}}$$

21 $${I_L} - {I_{in}}={I_{C1}}$$

22 $${I_{Lm}}= - {I_{Np}} - {I_{C1}}+{I_{D2}}$$

23 $${I_L}={I_{D2}}$$

24 $${I_{Lm}}+{I_{Np}}+{I_{C2}}={I_{D3}}$$

25 $${I_{Ns}}={I_{C2}}$$

26 $${I_{C3}}= - {I_O}+{I_{D3}}+{I_{D5}}$$

27 $$- {I_{Ns}}={I_{D5}}$$

28 $${I_{C4}}= - {I_O}+{I_{D5}}$$

29 $${I_{in}}={I_O}+{I_{C3}}$$

### Voltage gain calculation

The steady-state operation of the proposed converter in CCM is analyzed by studying the circuit behavior across each switching interval and evaluating the corresponding equivalent circuits, as illustrated in Fig. 2. By applying the volt–second balance principle to both the input inductor and the magnetizing inductance, the capacitor voltages can be determined, and the analytical relationship between the input and output voltages can be established. Based on the distinct operating modes, the following analytical expressions are derived.

30 $${V_{C1}}=\frac{{D{V_{in}}}}{{1 - D}}$$

31 $${V_{C2}}=\frac{{{V_{in}}(kn - Dkn+1)}}{{{{(1 - D)}^2}}}$$

32 $${V_{C3}}=\frac{{{V_{in}}}}{{{{(1 - D)}^2}}}$$

33 $${V_{C4}}=\frac{{{V_{in}}(kn+1)}}{{{{(1 - D)}^2}}}$$

The mathematical expression defining the output voltage of the suggested converter can be written as follows:

34 $${V_O}=\frac{{{V_{in}}(kn+2)}}{{{{(1 - D)}^2}}}$$

Under the assumption of ideal magnetic coupling in the coupled inductor (k = 1), the output voltage gain is given by the following expression:

35 $$G=\frac{{{V_O}}}{{{V_{in}}}}=\frac{{n+2}}{{{{(1 - D)}^2}}}$$

### Voltage stresses of semiconductors

The voltage stresses experienced by the power switches and diode components can be described by the following expressions:

36 $${V_{S1}}={V_{S2}}=\frac{{{V_{in}}}}{{{{(1 - D)}^2}}}$$

37 $${V_{D1}}=\frac{{D{V_{in}}}}{{{{(1 - D)}^2}}}$$

38 $${V_{D2}}=\frac{{{V_{in}}}}{{1 - D}}$$

39 $${V_{D3}}=\frac{{{V_{in}}}}{{{{(1 - D)}^2}}}$$

40 $${V_{D4}}={V_{D5}}=\frac{{{V_{in}}(kn+1)}}{{{{(1 - D)}^2}}}$$

### Current analysis of the components

Using the operating waveforms shown in Fig. 2, the current expressions of the switches and diodes in each operating mode are analytically derived and presented in the following Eq. 

41 $${I_{S1 - Mode1}}={I_{S2 - Mode1}}=\frac{{{I_O}(2D+n+1 - {D^2})}}{{2D{{(1 - D)}^2}}}$$

42 $${I_{D1 - Mode1}}={I_{D2 - Mode2}}=\frac{{{I_O}(n+2)}}{{{{(1 - D)}^2}}}$$

43 $${I_{D3 - Mode2}}={I_{D5 - Mode2}}=\frac{{{I_O}}}{{1 - D}}$$

44 $${I_{D4 - Mode1}}=\frac{{{I_O}}}{D}$$

The currents of the input inductor L and the magnetizing inductance L_m_​ are analytically expressed in Eqs. (45) and (46), respectively.

45 $${I_L}=\frac{{{I_O}(n+2)}}{{{{(1 - D)}^2}}}$$

46 $${I_{Lm}}=\frac{{{I_O}(2+n)}}{{1 - D}}$$

By analyzing the equivalent circuits of each operating interval and applying the ampere–second balance principle, the following expressions for the capacitor currents can be obtained:

47 $${I_{C1 - Mode1}}= - \frac{{{I_O}(2D+n)}}{{D(1 - D)}}$$

48 $${I_{C1 - Mode2}}=\frac{{{I_O}(2D+n)}}{{{{(1 - D)}^2}}}$$

49 $${I_{C2 - Mode1}}=\frac{{{I_O}}}{D}$$

50 $${I_{C2 - Mode2}}= - \frac{{{I_O}}}{{1 - D}}$$

51 $${I_{C3 - Mode1}}= - \frac{{(1+D){I_O}}}{D}$$

52 $${I_{C3 - Mode2}}=\frac{{(1+D){I_O}}}{{1 - D}}$$

53 $${I_{C4 - Mode1}}= - {I_O}$$

54 $${I_{C4 - Mode2}}=\frac{{D{I_O}}}{{1 - D}}$$

### Average currents of semiconductors

The mean current stresses imposed on the power switch and diode devices can be formulated as follows:

A precise evaluation of power losses in DC–DC converters necessitates the computation of root-mean-square (RMS) currents for all circuit elements. The procedures employed to calculate the RMS currents of individual components are provided in Eqs. (59)–(67) and are summarized below.

59 $${I_{S1,RMS}}={I_{S2,RMS}}=\frac{{{I_O}(2D+n+1 - {D^2})}}{{2\sqrt D {{(1 - D)}^2}}}$$

60 $${I_{D1,RMS}}=\frac{{{I_O}(n+2)\sqrt D }}{{{{(1 - D)}^2}}}$$

61 $${I_{D2,RMS}}=\frac{{{I_O}(2+n)}}{{\sqrt {{{(1 - D)}^3}} }}$$

62 $${I_{D3,RMS}}={I_{D5,RMS}}=\frac{{{I_O}}}{{\sqrt {1 - D} }}$$

63 $${I_{D4,RMS}}=\frac{{{I_O}}}{{\sqrt D }}$$

64 $${I_{C1,RMS}}=\frac{{{I_O}(2D+n)}}{{\sqrt D (1 - D)}}+\frac{{{I_O}(2D+n)}}{{\sqrt {{{(1 - D)}^3}} }}$$

65 $${I_{C2,RMS}}==\frac{{{I_O}}}{{\sqrt D }}+\frac{{{I_O}}}{{\sqrt {(1 - D)} }}$$

66 $${I_{C3,RMS}}=\frac{{{I_O}(1+D)}}{{\sqrt D }}+\frac{{{I_O}(1+D)}}{{\sqrt {1 - D} }}$$

67 $${I_{C4,RMS}}={I_o}\sqrt D +\frac{{{I_O}D}}{{\sqrt {1 - D} }}$$

### DCM operation of the proposed converter

In DCM operation, the proposed converter exhibits three distinct operating modes within one switching period. The first two operating modes are identical to those described for CCM operation. The key difference in DCM operation appears in the third operating mode, which is absent in CCM and occurs when the current in the magnetizing inductance of the coupled inductor (and consequently the current in the input inductor) falls to zero before the next switching cycle begins. This additional mode occupies a time interval denoted as D_x_​. During this interval, all semiconductor devices including the power switches and diodes are turned off, and there is no active energy transfer from the source or the inductive elements. The inductors are fully demagnetized, and the load is supplied only by the capacitors C_3_ and C_4_. This zero-current interval is the defining characteristic of DCM operation. The presence of this third mode modifies the current waveforms and slightly alters the voltage–current relationships compared to CCM; however, the overall operating principle of the converter remains consistent. Importantly, the existence of the D_x_​ interval provides additional flexibility under light-load or low-power conditions, where DCM naturally occurs. In such cases, the converter can still maintain proper voltage boosting behavior without instability, which demonstrates the robustness of the proposed topology.

#### Third switching subinterval

This phase starts when the currents in inductors L and Lm diminish to zero, causing the voltage across both inductors to drop to zero as well. Throughout this phase, all semiconductor components are switched off. The inductors are completely demagnetized, and the capacitors C_3_ and C_4_ solely provide power to the load. The related equation for this mode is expressed as.

68 $${V_O} - {V_{C3}}={V_{C4}}$$

The voltage equations for the capacitor can be obtained from the governing formulas, which are shown below:

69 $${V_{C1}}=\frac{{{V_{in}}D}}{{1 - D - {D_x}}}$$

70 $${V_{C2}}=\frac{{{V_{in}}(1 - {D_x})( - {D_x}+n - Dn+1 - {D_x}n)}}{{{{(1 - D - {D_x})}^2}}}$$

71 $${V_{C3}}=\frac{{{V_{in}}{{(1 - {D_x})}^2}}}{{{{(1 - D - {D_x})}^2}}}$$

72 $${V_{C4}}=\frac{{{V_{in}}{{(1 - {D_x})}^2}(1+n)}}{{{{(1 - D - {D_x})}^2}}}$$

The output voltage of the proposed circuit is represented by the following mathematical expression:

73 $${V_O}=\frac{{{V_{in}}{{(1 - {D_x})}^2}(n+2)}}{{{{(1 - D - {D_x})}^2}}}$$

The voltage gain can be expressed as:

74 $${G_{DCM}}=\frac{{{V_O}}}{{{V_{in}}}}=\frac{{{{(1 - {D_x})}^2}(n+2)}}{{{{(1 - D - {D_x})}^2}}}$$

### Efficiency calculation

This section is devoted to analyzing both conduction and switching losses of the proposed converter with the objective of enhancing its overall efficiency. The loss evaluation incorporates the effects of non-ideal parameters, including the internal resistances of the diodes (r_D_​), power switches (r_S_​), the input inductor (r_L_​), the magnetizing branch of the coupled inductor (r_Lm_​​), and the capacitors (r_C_​), as well as the forward voltage drops across the diodes (V_FD_) and the switch (V_FS_). Based on these non-ideal factors, the mathematical expressions describing conduction and switching losses are derived as follows:

75 $${P_{Conduction,Switches}}=\sum\limits_{{n=1}}^{2} {{\mathrm{(}}{{\mathrm{V}}_{{\mathrm{FSn}}}}} \times {{\mathrm{I}}_{Sn - average}})+({r_{Sn}} \times {{\mathrm{I}}_{Sn - RMS}}^{2})$$

76 $${P_{Conduction,Diodes}}=\sum\limits_{{n=1}}^{5} {{\mathrm{(}}{{\mathrm{V}}_{{\mathrm{FDn}}}}} \times {{\mathrm{I}}_{Dn - average}})+({r_{Dn}} \times {{\mathrm{I}}_{Dn - RMS}}^{2})$$

77 $$\left\{ \begin{gathered} {P_{Conduction,L}}=({r_L} \times {{\mathrm{I}}_L}^{2}) \hfill \\ {P_{Conduction,Lm}}=({r_{Lm}} \times {{\mathrm{I}}_{Lm}}^{2}) \hfill \\ \end{gathered} \right.$$

78 $${P_{Conduction,Capacitors}}=\sum\limits_{{n=1}}^{4} {{\mathrm{(}}{r_{{C_n}}} \times {{\mathrm{I}}_{{C_n} - RMS}}^{2})}$$

79 $${P_{Switching,Switches}}=\sum\limits_{{n=1}}^{2} {{\mathrm{(}}\frac{1}{6} \times {f_{\mathrm{s}}} \times {{\mathrm{V}}_{{\mathrm{Sn}}}}} \times {{\mathrm{I}}_{Sn}} \times ({t_{ON}}+{t_{OFF}}))$$

80 $$\left\{ \begin{gathered} {P_{Switching,{D_1}}}=\frac{1}{6} \times {f_{\mathrm{s}}} \times {{\mathrm{V}}_{{D_1}}} \times {I_{rr}} \times {t_b} \hfill \\ {P_{Switching,{D_2}}}=\frac{1}{6} \times {f_{\mathrm{s}}} \times {{\mathrm{V}}_{{D_2}}} \times {I_{rr}} \times {t_b} \hfill \\ {P_{Switching,{D_4}}}=\frac{1}{6} \times {f_{\mathrm{s}}} \times {{\mathrm{V}}_{{D_4}}} \times {I_{rr}} \times {t_b} \hfill \\ {P_{Switching,{D_5}}}=\frac{1}{6} \times {f_{\mathrm{s}}} \times {{\mathrm{V}}_{{D_5}}} \times {I_{rr}} \times {t_b} \hfill \\ \end{gathered} \right.$$

The overall power loss associated with both conduction and switching phenomena in the switches and diodes can be represented as follows:

82 $${P_{D,Tot}}={P_{Cond,{D_{1,2,3,4,5}}}}+{P_{SW,{D_{1,2,4,5}}}}$$

The expression employed to determine the total power dissipation in the capacitors is given by:

83 $${P_{C,Tot}}={P_{Cond,{C_1}}}+{P_{Cond,{C_2}}}+{P_{Cond,{C_3}}}+{P_{Cond,{C_4}}}$$

The power dissipation occurring within the inductor core is calculated using the following relation:

84 $${P_C}=kf_{s}^{\alpha }B_{m}^{\beta }$$

Inductor core losses (PC) are commonly specified in terms of power dissipation per unit mass, expressed in watts per kilogram (W/kg). These losses are typically evaluated using the Steinmetz equation, which is based on three empirically determined coefficients α, β, and k collectively referred to as the Steinmetz parameters. For each magnetic material, these parameters are generally provided by the core manufacturer. In the case of ferrite magnetic cores, the exponent α usually lies between 1 and 2. By incorporating Faraday’s law, the corresponding expression for estimating the magnetic core loss of the inductor can therefore be formulated as follows:

85 $${V_L}=N\frac{{d\varphi (t)}}{{dt}}=N{A_c}\frac{{dB(t)}}{{dt}}$$

The term Ac denotes the effective cross-sectional area of the magnetic core, which is generally specified by the core manufacturer. Using this parameter, the peak change in magnetic flux density (ΔB) within the inductors can be expressed as follows:

86 $$\Delta B=\frac{1}{{N{A_c}}}\int\limits_{0}^{{D{T_s}}} {{V_{in}}dt}$$

For inductive elements, the total power dissipated in the magnetic core is calculated by multiplying the specific core loss by the mass of the core, expressed as P_Core_​=PC​M, where M represents the mass of the magnetic material. By assuming that the peak flux density is equal to B_m_ = ΔB/2, the corresponding expression used to estimate the core loss can be formulated as follows:

87 $${P_{Core}}=kf_{s}^{\alpha }{\left( {\frac{{{V_{in}}D}}{{2{N_P}{A_c}{f_s}}}} \right)^\beta }M$$

An ETD 49/25/16 ferrite core is selected for the inductors, characterized by Steinmetz coefficients k = 4.124 × 10^− 5^, α = 1.72, and β = 2.76. Based on these parameters, the total power dissipation of the proposed high step-up converter can be formulated as follows:

88 $${P_{Loss}}={P_{S,Tot}}+{P_{D,Tot}}+{P_{Cond,Lm}}+{P_{Cond,L}}+{P_{Core}}+{P_{C,Tot}}$$

Accordingly, the overall efficiency (η) of the proffered circuit is specified as follows:

89 $$\eta =\frac{{{P_{Out}}}}{{{P_{Out}}+{P_{Loss}}}}$$

**Fig. 4:**
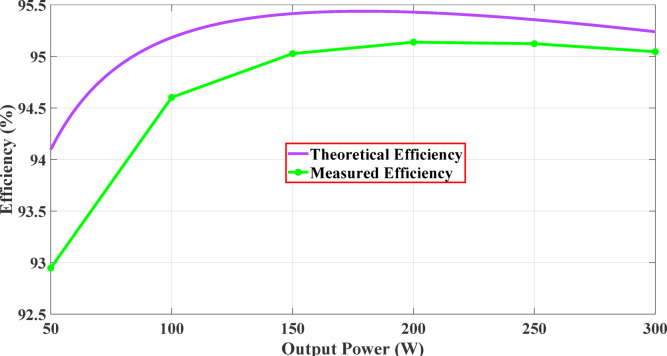
A comprehensive comparison between the analytically estimated efficiency and the experimentally measured efficiency of the proposed boost converter over a range of output power levels.

**Fig. 5:**
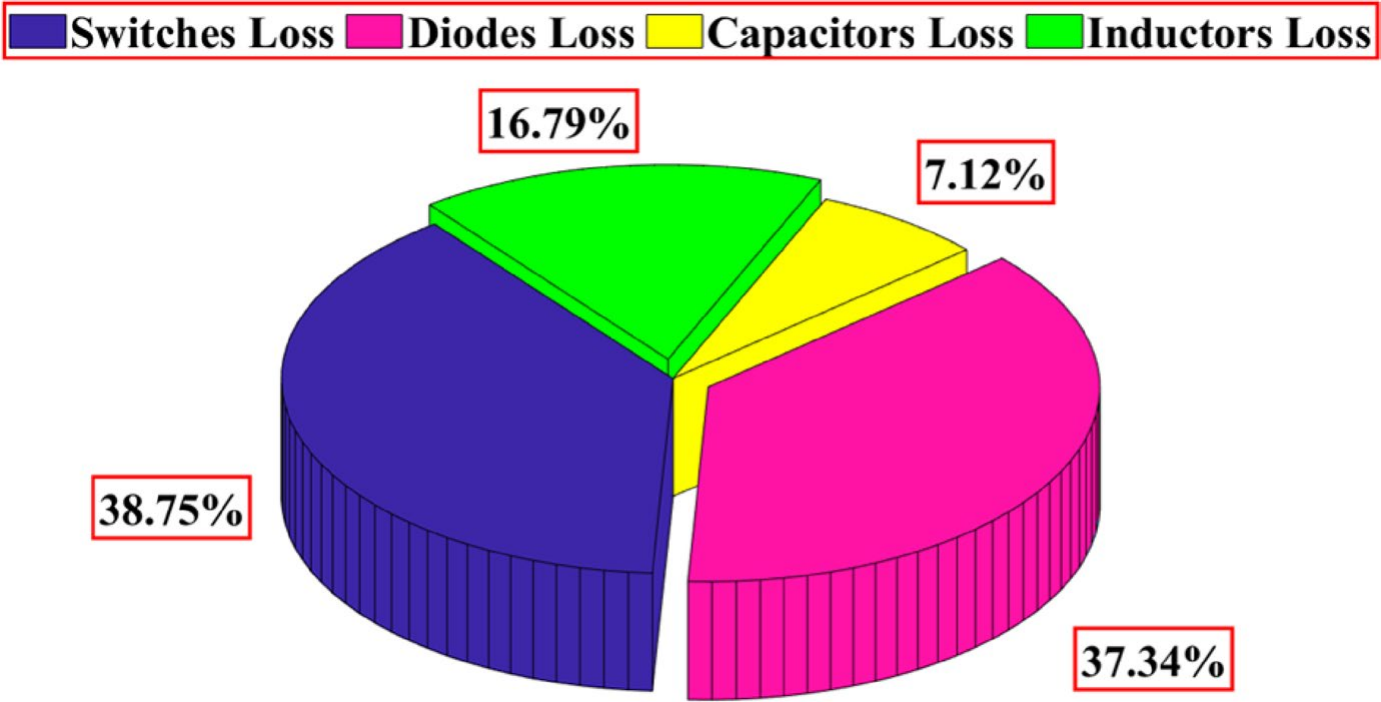
The relative contribution of switches, diodes, capacitors, and inductors to the total power loss of the converter.

Figure 4 presents a comparison between the calculated efficiency of the proposed converter and the experimentally obtained efficiency across different output power levels. The analytical efficiency curve is generated using the formulations given in Eqs. (75)–(89). In addition, Fig. 5 illustrates the breakdown of power losses among the converter components, revealing that the dominant share of losses arises from the power switches and diodes, whereas the capacitors and inductors contribute only a minor portion of the total losses.

### Key parameter design guidance

#### Capacitors voltage ripple in boost mode

The required capacitance values are obtained by evaluating the average currents passing through each capacitor over all operating intervals, while also taking into account the capacitor voltage levels, the converter duty ratio, the permissible voltage ripple (x_C_%), and the fixed switching frequency of 50 kHz. Based on these design considerations, the minimum capacitance values for C_1_​, C_2_​, C_3_​, and C_4_​ can be determined as follows:

90 $${C_1} \geqslant \frac{{{I_O} \times (2D+n)}}{{{f_s} \times D \times {V_{in}} \times {x_{C1}}\% }}$$

91 $${C_2} \geqslant \frac{{{I_O} \times {{(1 - D)}^2}}}{{{f_s} \times {V_{in}}(kn - Dkn+1) \times {x_{C2}}\% }}$$

92 $${C_3} \geqslant \frac{{{I_O} \times (1+D){{(1 - D)}^2}}}{{{f_s} \times {V_{in}} \times {x_{C3}}\% }}$$

93 $${C_4} \geqslant \frac{{{I_O} \times D{{(1 - D)}^2}}}{{{f_s} \times (kn+1) \times {V_{in}} \times {x_{C4}}\% }}$$

### Inductor design

Determining suitable values for the input inductor L and the magnetizing inductance L_m_​ of the coupled inductor involves evaluating several critical design factors. These include the average current carried by each inductor, the voltages applied during different operating intervals, the converter duty ratio, the permissible current ripple margins (X_L_% for L and X_Lm_​​% for L_m_​), and the switching frequency. Considering these parameters, the minimum inductance values required for L and L_m_​ can be formulated as follows:

94 $$L \geqslant \frac{{{V_{in}} \times D \times {{(1 - D)}^2}}}{{{f_s} \times {\mathrm{Io}}{\mkern 1mu} \times \left( {{\mkern 1mu} n+2} \right) \times {x_L}\% }}$$

95 $${L_m} \geqslant \frac{{k \times {V_{in}} \times D}}{{{f_s} \times {\mathrm{Io}} \times {\mkern 1mu} \left( {n+2} \right) \times {x_{Lm}}\% }}$$

### Small-signal modeling

A suitable pole-placement control approach is adopted to guarantee stable regulation of the output voltage. The design of an effective closed-loop controller requires the formulation of a small-signal model of the converter. Therefore, the system equations for the two switching subintervals are derived and presented as follows:

96 $$\left[ {\begin{array}{*{20}{c}} {{{\dot {I}}_L}} \\ {{{\dot {I}}_{Lm}}} \\ {{{\dot {V}}_{C1}}} \\ \begin{gathered} {{\dot {V}}_{C2}} \hfill \\ {{\dot {V}}_{C3}} \hfill \\ \end{gathered} \\ {{{\dot {V}}_{C4}}} \end{array}} \right]=[{A_X}]\left[ {\begin{array}{*{20}{c}} {{I_L}} \\ {{I_{Lm}}} \\ {{V_{C1}}} \\ \begin{gathered} {V_{C2}} \hfill \\ {V_{C3}} \hfill \\ \end{gathered} \\ {{V_{C4}}} \end{array}} \right]+[{B_X}]{V_{in}}$$

Where X = 1, 2.

By employing state-space averaging and ignoring higher-order perturbation terms, a small-signal model of the proposed converter is derived.

97 $$\left\{ \begin{gathered} \dot {\tilde {x}}=A\tilde {x}+B\tilde {u} \hfill \\ y=C\tilde {x}+D\tilde {u} \hfill \\ \end{gathered} \right.$$

The mathematical expressions describing the system states ($\tilde {x}$), control inputs ($\tilde {u}$), and the output (y) are specified as follows:

98 $${\tilde {x}^T}=\left[ {\begin{array}{*{20}{c}} {{{\tilde {i}}_L}}&{{{\tilde {i}}_{Lm}}}&{{{\tilde {v}}_{C1}}}&{{{\tilde {v}}_{C2}}}&{{{\tilde {v}}_{C3}}}&{{{\tilde {v}}_{C4}}} \end{array}} \right]$$

99 $$\tilde {u}=\left[ {\tilde {d}} \right]$$

100 $${y^T}=\left[ {{I_L}} \right]$$

Under the pole-placement control methodology, the locations of the closed-loop poles can be arbitrarily selected as long as the system is completely controllable in the state-space representation. Accordingly, the controllability matrix of the proposed converter is formulated as follows:

101 $${\Phi _C}=\left[ {B \vdots AB \vdots {A^2}B \vdots \cdots \vdots {A^{n - 1}}B} \right]$$

A system is regarded as fully controllable when the rank of its controllability matrix is equal to five, corresponding to the number of state variables. To ensure this condition is met, two supplementary integral state variables are defined as follows:

102 $$\dot {q}(t)=r(t) - y(t)=r(t) - {\tilde {i}_L}(t)$$

After incorporating the integral state variables, the state-space representation and the output equations are updated as follows:

103 $$\begin{gathered} \left[ {\begin{array}{*{20}{c}} {\dot {\tilde {x}}(t)} \\ \cdots \\ {\dot {q}(t)} \end{array}} \right]=\left[ {\begin{array}{*{20}{c}} A& \vdots &0 \\ \cdots & \vdots & \cdots \\ { - C}& \vdots &0 \end{array}} \right]\left[ {\begin{array}{*{20}{c}} {\tilde {x}(t)} \\ \cdots \\ {q(t)} \end{array}} \right]+\left[ {\begin{array}{*{20}{c}} B \\ \cdots \\ 0 \end{array}} \right]\tilde {u}(t)+\left[ {\begin{array}{*{20}{c}} 0 \\ \cdots \\ I \end{array}} \right]r(t) \hfill \\ y(t)=\left[ {\begin{array}{*{20}{c}} C& \vdots &0 \end{array}} \right]\left[ {\begin{array}{*{20}{c}} {\tilde {x}(t)} \\ \cdots \\ {q(t)} \end{array}} \right] \hfill \\ \end{gathered}$$

**Fig. 6:**
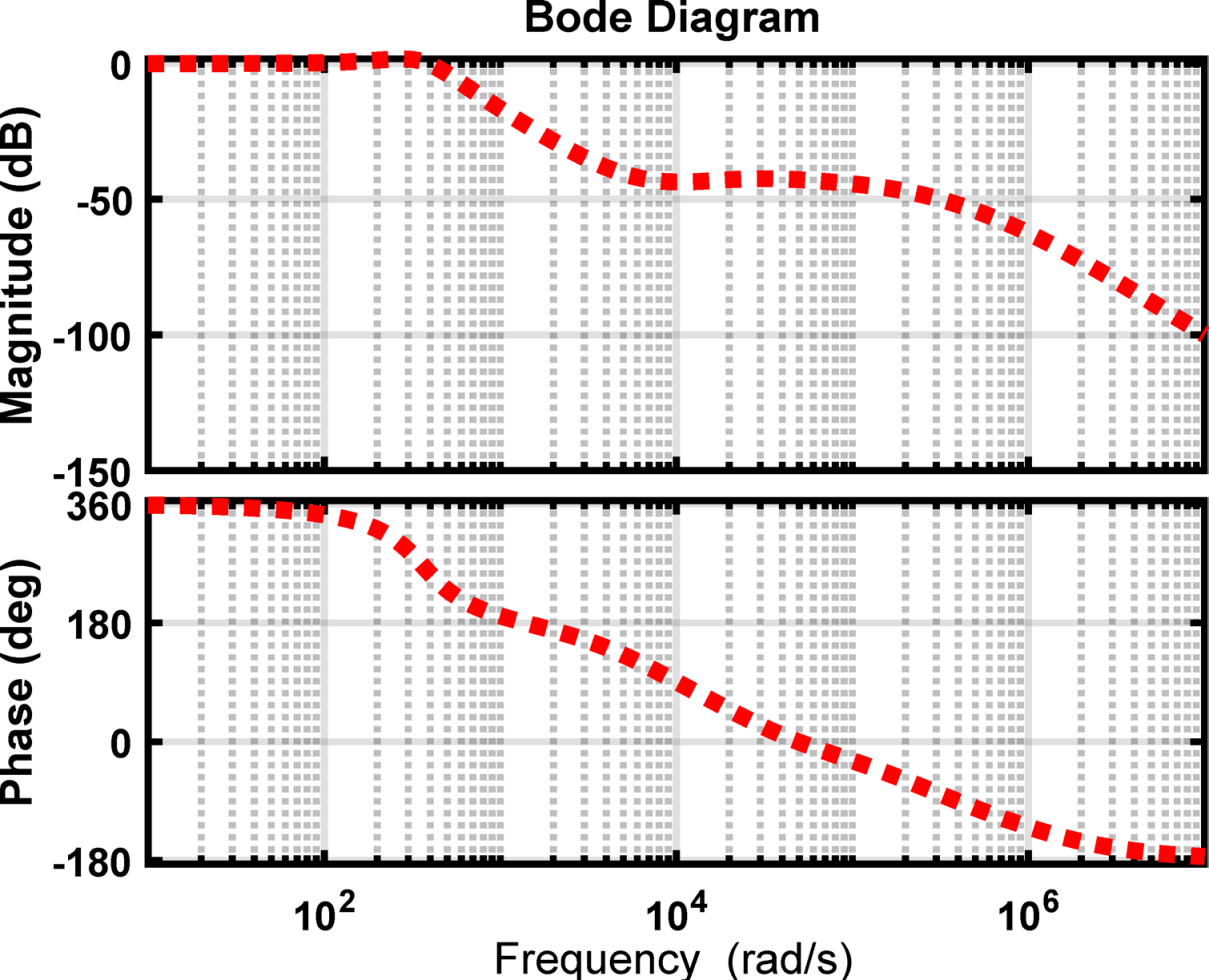
Bode plot of the transfer function corresponding to the input inductor current i_L_​.

**Fig. 7:**
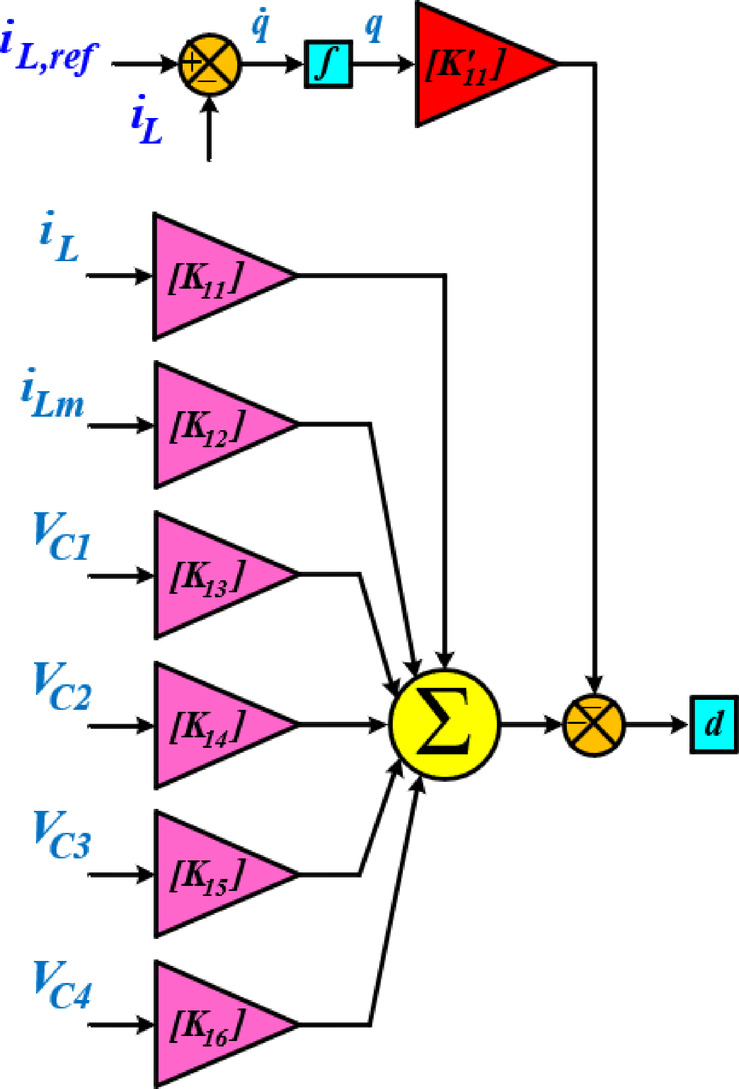
Current control loop for the inductor L.

To achieve the desired stability requirements—namely, a gain margin (GM) of at least 10 and a phase margin (PM) within the range of 60° to 80° a trial-and-error tuning procedure is employed to appropriately place the closed-loop poles. Based on this approach, the Bode diagram of the control strategy for the proposed converter is presented in Fig. 6. As illustrated, the gain margin associated with the magnetizing inductor current exceeds the specified threshold, with GM (i_Lm_​​) > 10. Moreover, the phase margin of the closed-loop current control path for i_Lm_​​ is calculated to be 77.074°, which lies well within the acceptable stability range. Figure 7 illustrates the current regulation loop for inductor L.

### Comparison study

A thorough comparative study was performed to demonstrate the advantages of the proposed converter. This assessment includes sixteen competing topologies reported between 2023 and 2025. Table 1 presents a detailed, feature-by-feature comparison between the proposed design and these alternatives, considering key performance indicators such as voltage gain, maximum switch voltage stress, rated output power, efficiency, total number of devices, and the availability of a common ground between input and output. The converters included in this evaluation are drawn from the literature cited in references^8–23^.

**Fig. 8:**
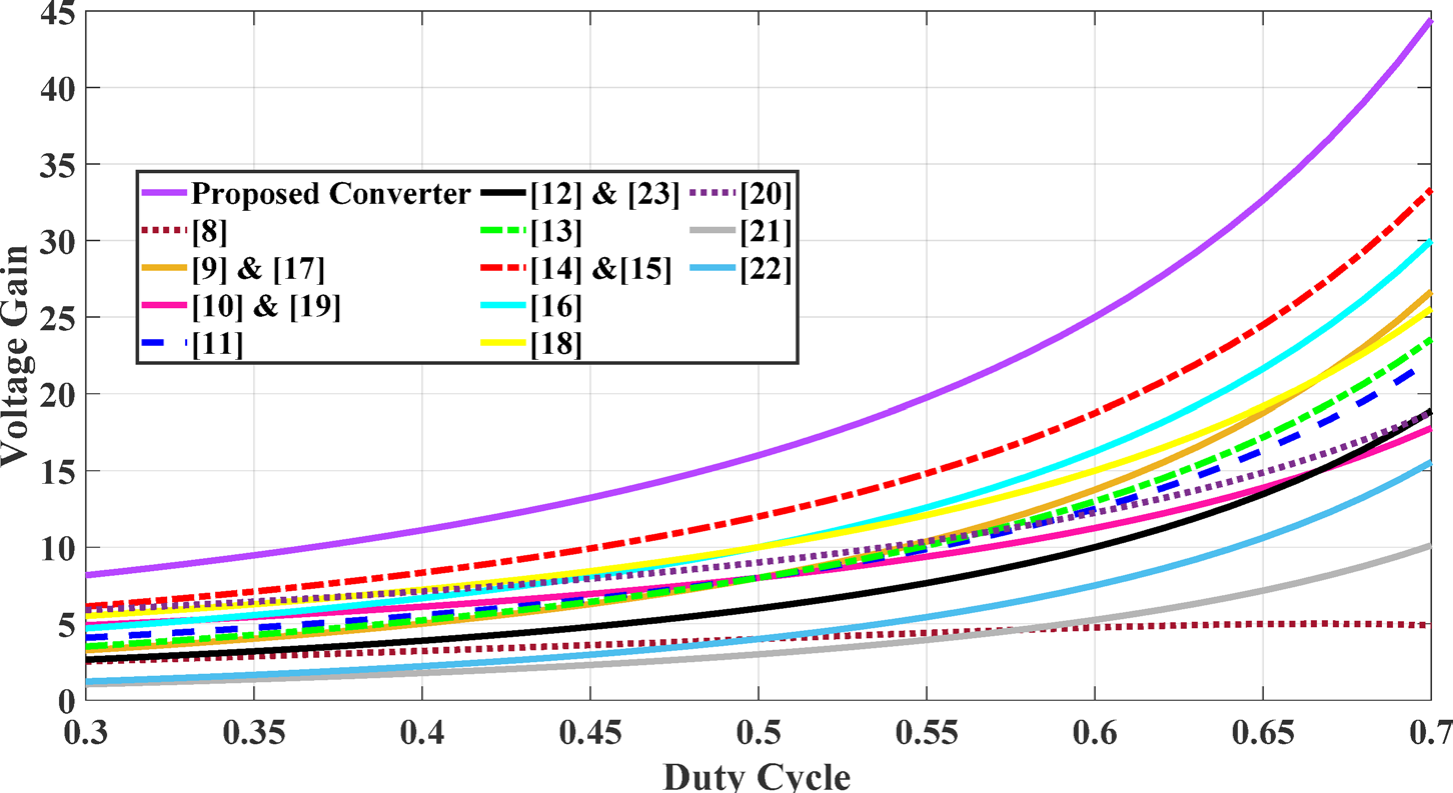
Duty-cycle–dependent variation of output voltage gain for the compared high step-up converter topologies.

Since the primary objective of this work is to achieve ultra–high step-up capability, voltage gain is selected as the main performance criterion. Based on the data summarized in Table 1; Fig. 8 illustrates the variation of voltage gain with duty cycle for all reviewed boost converter structures. The results clearly indicate that the proposed topology delivers the highest voltage gain among all the designs considered in Table 1.

**Fig. 9:**
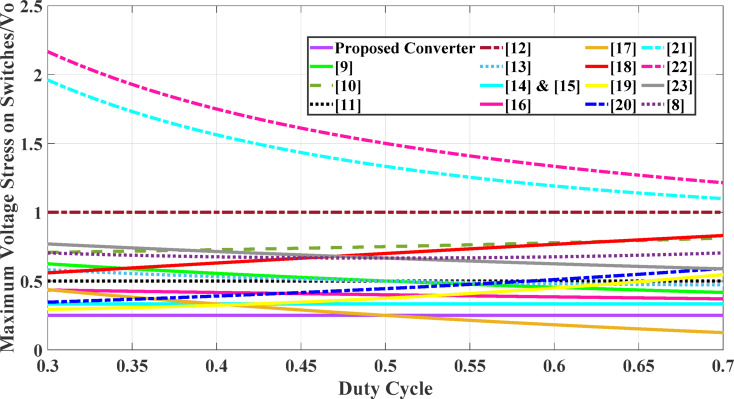
Comparison of peak switch voltage stress versus duty cycle.

The maximum voltage stress experienced by the switches is listed in the second column of Table 1, while its dependence on duty cycle is illustrated in Fig. 9. Across most operating conditions, the proposed converter demonstrates superior management of peak switch voltage stress compared with alternative topologies. A single exception is observed for the converter reported in^17^, which exhibits lower switch stress than the proposed design when the duty cycle exceeds 50%. However, for duty cycles below 50%, the proposed topology achieves the lowest switch voltage stress among all compared configurations. It is worth noting that operation at duty cycles below 50% is generally preferable, as it enhances converter performance and reduces losses due to the lower duty ratio. An additional benefit of the proposed design is the reduced voltage across the switches, which allows the use of smaller inductive components during implementation.

The third column of Table 1 summarizes the rated power and efficiency of each converter. The proposed topology is designed for a 300 W output and achieves an efficiency of 95.24%, as experimentally validated in Fig. 4. In contrast, most of the competing designs listed in Table 1 are rated for output powers below 300 W. Even when operating at its full rated power, the proposed converter maintains its high efficiency, outperforming many of the other topologies. Notably, several competing designs operate at lower power levels yet still exhibit inferior efficiency performance.

**Fig. 10:**
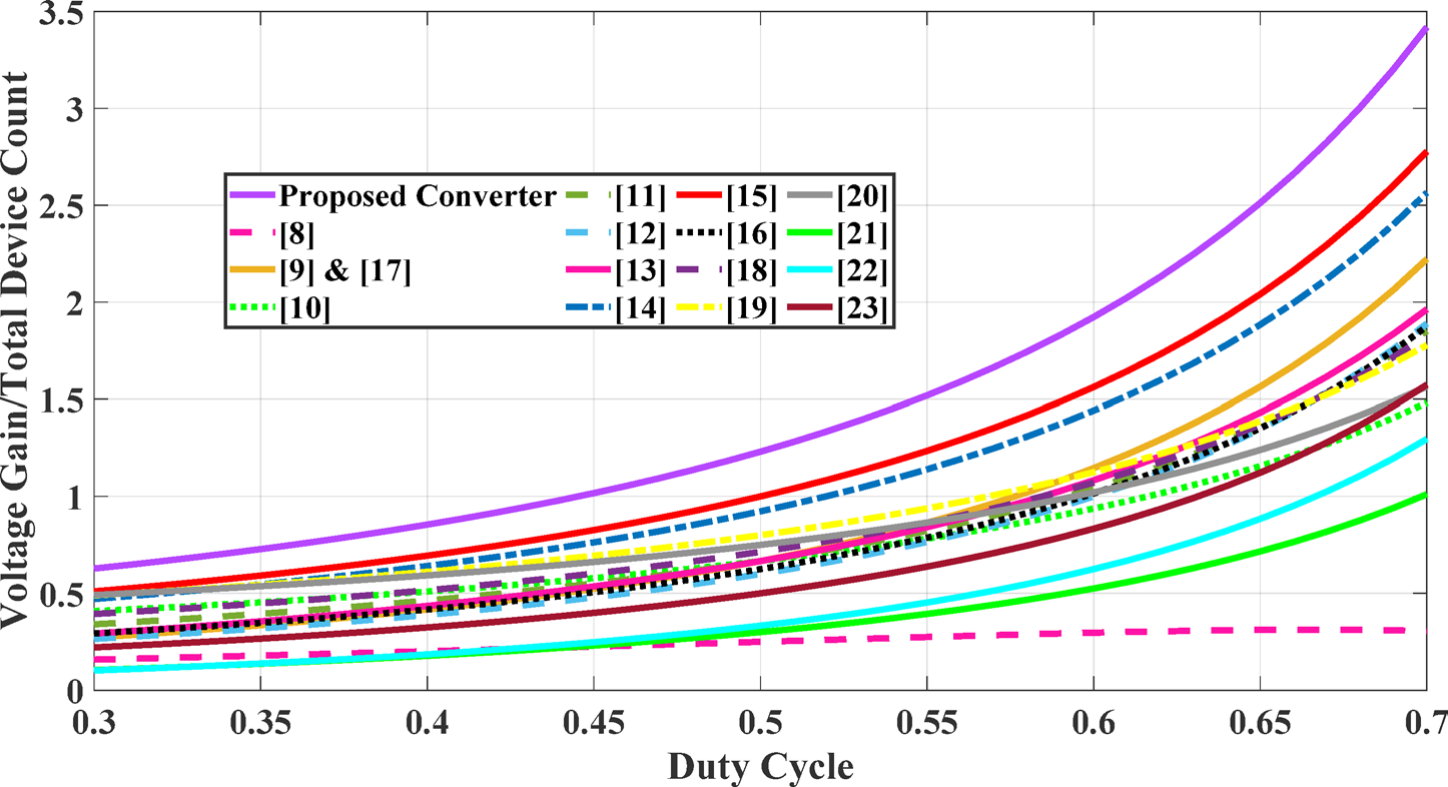
Voltage gain–to–total device count ratio evaluated over different duty-cycle values.

Component utilization is reported in the fourth column of Table 1, while the final column provides the total device count along with a detailed breakdown. An optimal high-gain converter should minimize the number of components while maximizing voltage gain. To evaluate this balance, Fig. 10 plots voltage gain as a function of total device count for all examined topologies. The results show that the proposed converter achieves the highest voltage gain per device count, indicating a clear cost-effectiveness advantage for comparable performance.

The final aspect of the comparison examines whether a common ground is shared between the input and output, a critical feature since converters lacking a common ground are generally more susceptible to electromagnetic interference, which can adversely affect performance. Unlike the converters reported in^12,13,19,20^, and^22^, the proposed topology provides a common ground connection. Also, the input current continuity factor has been examined in the comparison table; maintaining a continuous input current is highly suitable and important for renewable energy applications. The proposed converter offers a significant advantage in terms of minimum inductor size compared to the converters in references^8] and [9^. By combining a quadratic structure with a coupled inductor, it reduces the required volt–second stress and current ripple, allowing the use of smaller inductors without compromising the voltage gain. This leads to a more compact design, enhanced power density, and reduced material costs, making it a more practical solution for high-voltage gain applications.

In summary, the comparative evaluation underscores the key strengths of the proposed converter, including its superior voltage gain, reduced switch voltage stress, outstanding gain-to-device-count ratio across duty cycles, and high efficiency at the 300 W power level.

### Experimental results

**Fig. 11:**
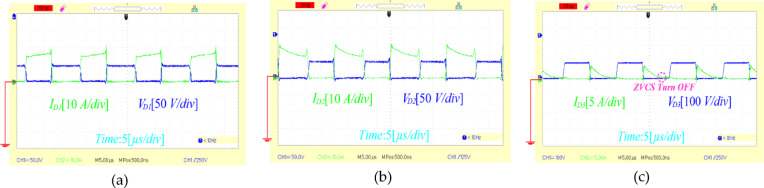
Photographs of experimental prototype.

**Fig. 12:**
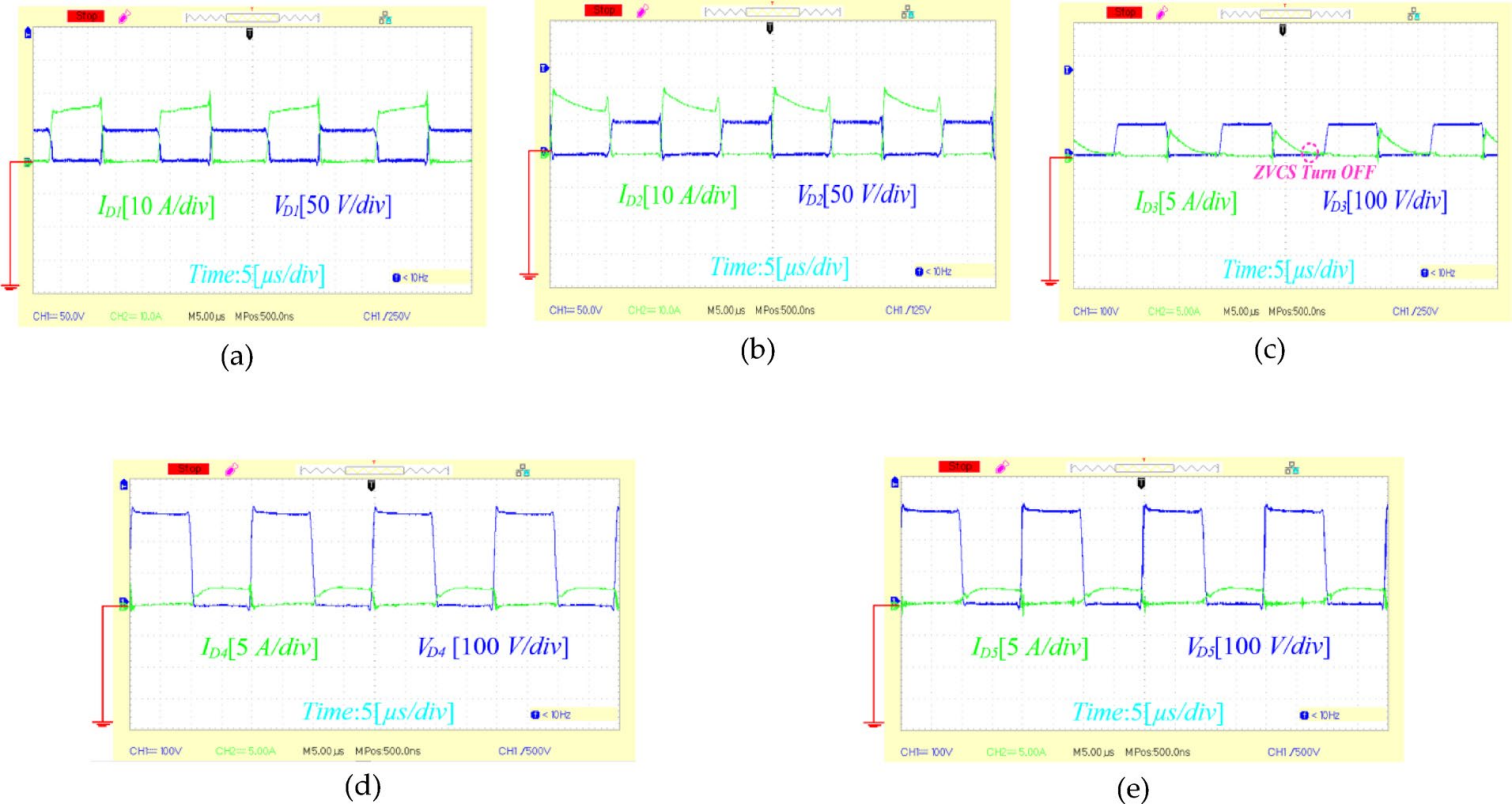
The experimental waveforms of output port and power switches, **(a)** voltage and current of the output port, **(b)** voltage and current of the switch S_1_, **(c)** voltage and current of the switch S_2_.

To verify the accuracy of the proposed design and evaluate its practical performance, a 300 W laboratory prototype was developed for operation with a 25 V input voltage and a 400 V output voltage. Figure 11 shows images of the experimental prototype. The detailed specifications of the prototype are summarized in Table 2. High-frequency measurements were carried out using a current probe (MICSIG CP2100A, 800 kHz bandwidth) along with a voltage probe to record the voltage and current waveforms of the critical circuit components. As illustrated in Fig. 12(a), the prototype delivers an output voltage of 390 V at an output current of 0.77 A. Based on Eq. (35), the theoretical output voltage is calculated to be 400 V, showing close agreement with the experimental measurement.

Figure 12(b) presents the voltage and current waveforms of switch S₁, demonstrating that during the first operating mode it is subjected to only a small portion of the output voltage approximately 25%. This reduced voltage stress contributes significantly to lower switching losses. The conduction behavior of switch S₂ during the first switching interval is shown in Fig. 12(c). The experimental waveforms in Figs. 12(b) and 11(c) closely correspond to the analytical results obtained from Eqs. (36) and (41), indicating strong consistency between theoretical modeling and experimental validation.

**Fig. 13:**
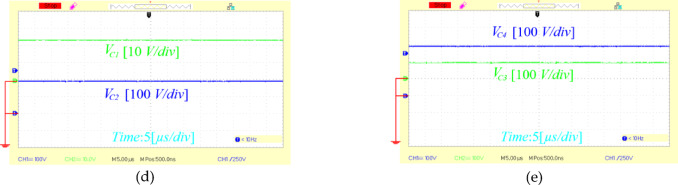
The experimental waveforms of diodes, (**a**) voltage and current of the diode D_1_, **(b)** voltage and current of the diode D_2_, **(c)** voltage and current of the diode D_3_, **(d**) voltage and current of the diode D_4_, **(e**) voltage and current of the diode D_5_.

The voltage and current characteristics of diodes D₁ and D₂ are depicted in Figs. 13(a) and 12(b), respectively. The measured values are in good agreement with the analytical predictions derived from Eqs. (37), (38), and (42). Furthermore, the operating behavior of diodes D₃ and D₄ is illustrated in Figs. 13(c) and 12(d). In particular, diode D₃ exhibits zero-voltage current switching during turn-off, as expected from the theoretical analysis. These observations align well with the predictions of Eqs. (39), (40), (43), and (44), confirming the validity of the analytical model.

**Table 1:** Comparison of the proposed configuration with other topologies.

Ref/PY*	Voltage gain	Maximum voltage stress on Switches	Power (W) /Efficiency	No. of components					Total component count /Common ground/ Continuous input current
				S	D	C	L	CI	
^8^	$\frac{{1+2D - 2{D^2}}}{{{{(1 - D)}^2}}}$	$\frac{{{V_o}}}{{1+2D - 2{D^2}}}$	200/90%	1	5	6	4	0	16/Yes/Yes
^9^	$\frac{{n - 1+nD}}{{{{(1 - D)}^2}}}$	$\frac{{(n - 1){V_o}}}{{n - 1+nD}}$	100/93%	1	4	4	2	1	12/Yes/Yes
^10^	$\frac{{3 - 2D}}{{{{(1 - D)}^2}}}$	$\frac{{(2 - D){V_o}}}{{3 - 2D}}$	400/95.1%	1	5	4	1	1	12/Yes/Yes
^11^	$\frac{2}{{{{(1 - D)}^2}}}$	$\frac{{{V_o}}}{2}$	42/93.4%	1	5	4	0	2	12/Yes/Yes
^12^	$\frac{{1+D}}{{{{(1 - D)}^2}}}$	${V_O}$	500/94.3%	2	3	3	2	0	10/No/Yes
^13^	$\frac{{(1+3D - 2{D^2})}}{{{{(1 - D)}^2}}}$	$\frac{{{V_O}}}{{1+3D - 2{D^2}}}$	100/96%	2	3	4	3	0	12/No/Yes
^14^	$\frac{{n+1}}{{{{(1 - D)}^2}}}$	$\frac{{{V_O}}}{{n+1}}$	360/95.2%	1	5	5	0	2	13/ Yes/Yes
^15^	$\frac{{n+1}}{{{{(1 - D)}^2}}}$	$\frac{{{V_O}}}{{n+1}}$	400/95%	1	4	4	2	1	12/ Yes/Yes
^16^	$\frac{{2+D}}{{{{(1 - D)}^2}}}$	$\frac{{{V_O}}}{{2+D}}$	50/87%	1	6	6	3	0	16/ Yes/Yes
^17^	$\frac{{2D+n - 1}}{{{{(1 - D)}^2}}}$	$\frac{{(1 - D){V_o}}}{{2D+n - 1}}$	210/93.4%	2	4	4	1	1	12/Yes/Yes
^18^	$\frac{{3 - D}}{{{{(1 - D)}^2}}}$	$\frac{{(1+2D - {D^2}){V_o}}}{{3 - D}}$	300/94.5%	1	6	4	3	0	14/Yes/Yes
^19^	$\frac{{3 - 2D}}{{{{(1 - D)}^2}}}$	$\frac{{(1 - 2D{{(1 - D)}^2}){V_o}}}{{3 - 2D}}$	300/93.6%	2	3	3	2	0	10/No/Yes
^20^	$\frac{{{{(2 - D)}^2}}}{{{{(1 - D)}^2}}}$	$\frac{{{V_o}}}{{{{(2 - D)}^2}}}$	400/93.3%	2	4	4	2	0	12/No/Yes
^21^	$\frac{{D(2 - D)}}{{{{(1 - D)}^2}}}$	$\frac{{{V_O}}}{{D(2 - D)}}$	72/95%	2	2	3	3	0	10/ Yes/Yes
^22^	$\frac{{2D}}{{{{(1 - D)}^2}}}$	$\frac{{(1+D){V_O}}}{{2D}}$	60/97%	2	3	4	3	0	12/ No/Yes
^23^	$\frac{{1+D}}{{{{(1 - D)}^2}}}$	$\frac{{{V_O}}}{{1+D}}$	100/94%	1	4	4	3	0	12/ Yes/Yes
PC	$\frac{{kn+2}}{{{{(1 - D)}^2}}}$	$\frac{{{V_O}}}{{kn+2}}$	300/95.24%	2	5	4	1	1	13/Yes/Yes

*Number of Switches/Diodes/Capacitors/Inductors/Coupled Inductors.

**Table 2:** Specifications of the components used in the implemented converter.

Parameters	Values
Rated power (*P*_*o*_)	300 *W*
Input voltage	25 V
Output voltage	400 V
Switching Frequency (*f*_*s*_)	50 kHz
Turns Ratio *n* (*N*_*S*_*/N*_*P*_)	2
Input Inductor (*L*)	400 *µH*
Magnetizing Inductor (*L*_*m*_)	300 *µH*
Leakage Inductor (*L*_*k*_)	2 *µH*
Power Switches	IRFP260n
Diodes (*D*_*1*_, *D*_*2*_)	MBR30300CT
Diodes (*D*_*3*_, *D*_*4*_, *D*_*5*_)	Mur1560G
Cores type	ETD 49/25/16
Capacitor (*C*_*1*_)	68 µF/100V
Capacitor (*C*_*2*_, *C*_*4*_)	68 µF/350V
Capacitor (*C*_*3*_)	68 µF/200V

The performance of diode D₅, shown in Fig. 13(e), also closely matches the theoretical expectations derived from Eqs. (40) and (43), further reinforcing the agreement between measured and calculated results.

**Fig. 14:**
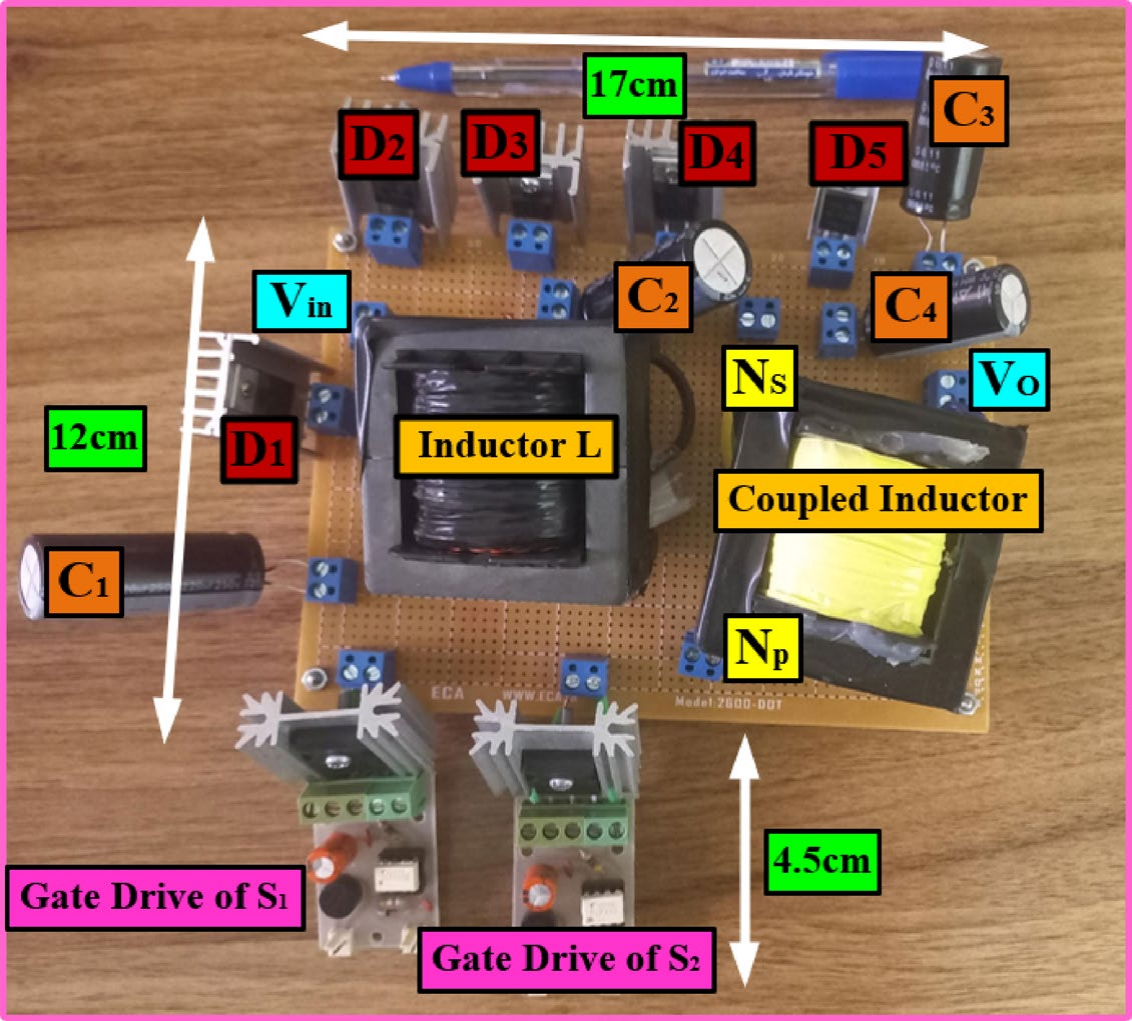
The experimental waveforms of diodes and capacitors, (a) voltage across the capacitors C_1_ and C_2_, (b) voltage across the capacitors C_3_ and C_4_.

Similarly, Eqs. (30) and (31) predict voltage levels of 25 V and 200 V across capacitors C₁ and C₂, respectively. Experimental measurements show voltages of approximately 23 V across C₁ and 193 V across C₂, as presented in Fig. 14(a), demonstrating a strong correlation with theoretical estimates. In addition, Eqs. (32) and (33) forecast voltages of 100 V across C₃ and 300 V across C₄. The corresponding experimental results, shown in Fig. 14(b), indicate measured voltages of 96 V and 294 V across C₃ and C₄, respectively, further confirming the close agreement between analytical predictions and experimental observations.

## Conclusions

This paper introduces an advanced quadratic high-gain DC–DC converter that employs a coupled-inductor mechanism to achieve substantial voltage amplification. By appropriately designing the turns ratio of the coupled inductor, the proposed topology attains exceptionally high voltage gain using a simple and efficient structural configuration. One of the key merits of the converter is the significantly reduced voltage stress across the active power switches compared with many existing high-gain converter topologies. This characteristic allows the implementation of low-voltage-rated semiconductor devices with lower on-state resistance, thereby decreasing conduction losses and improving conversion efficiency. The proposed converter exhibits several attractive features, including a high step-up voltage capability with negligible power loss, zero-voltage–current switching operation for diode D_3_, and enhanced controllability of the voltage gain through both duty-cycle regulation and adjustment of the coupled-inductor turns ratio. In contrast to conventional Z-source and quasi-Z-source converters, the proposed structure is free from duty-cycle constraints and supports operation over the entire range from 0 to 1. This extended operating range enables the realization of extremely large voltage gains at relatively low duty-cycle values, which substantially reduces conduction losses in the switching elements and further enhances the overall system efficiency.

## Data Availability

All data generated or analyzed during this study are included in this published article.
